# DNA methylation profile of triple negative breast cancer-specific genes comparing lymph node positive patients to lymph node negative patients

**DOI:** 10.1038/srep33435

**Published:** 2016-09-27

**Authors:** Andrea Mathe, Michelle Wong-Brown, Warwick J. Locke, Clare Stirzaker, Stephen G. Braye, John F. Forbes, Susan J. Clark, Kelly A. Avery-Kiejda, Rodney J. Scott

**Affiliations:** 1 Centre for Information Based Medicine, Hunter Medical Research Institute, NSW, 2305, Australia; 2 Priority Research Centre for Cancer, School of Biomedical Sciences and Pharmacy, Faculty of Health, University of Newcastle, NSW, 2308, Australia; 3 Epigenetics Research Program, Genomics and Epigenetics Division, Garvan Institute of Medical Research, Sydney, NSW, 2010, Australia; 4 Faculty of Medicine, St Vincent’s Clinical School, UNSW, NSW 2052 & St Vincent’s Hospital, Sydney, NSW, 2010, Australia; 5 Pathology North, John Hunter Hospital, Newcastle, NSW, 2305, Australia; 6 Department of Surgical Oncology, Calvary Mater Newcastle Hospital, Australian New Zealand Breast Cancer Trials Group, and School of Medicine and Public Health, University of Newcastle, NSW, 2308, Australia

## Abstract

Triple negative breast cancer (TNBC) is the most aggressive breast cancer subtype with no targeted treatment available. Our previous study identified 38 TNBC-specific genes with altered expression comparing tumour to normal samples. This study aimed to establish whether DNA methylation contributed to these expression changes in the same cohort as well as disease progression from primary breast tumour to lymph node metastasis associated with changes in the epigenome. We obtained DNA from 23 primary TNBC samples, 12 matched lymph node metastases, and 11 matched normal adjacent tissues and assayed for differential methylation profiles using Illumina HumanMethylation450 BeadChips. The results were validated in an independent cohort of 70 primary TNBC samples. The expression of 16/38 TNBC-specific genes was associated with alteration in DNA methylation. Novel methylation changes between primary tumours and lymph node metastases, as well as those associated with survival were identified. Altered methylation of 18 genes associated with lymph node metastasis were identified and validated. This study reveals the important role DNA methylation plays in altered gene expression of TNBC-specific genes and lymph node metastases. The novel insights into progression of TNBC to secondary disease may provide potential prognostic indicators for this hard-to-treat breast cancer subtype.

Breast cancer is responsible for the highest cancer incidence in women worldwide and its rates are increasing. Out of all breast cancer subtypes, triple negative breast cancer (TNBC) is the most aggressive, accounting for somewhere between 10 and 20% of all diagnosed breast cancers^1^,^2^,^3^. TNBC is recognised for its more frequent and rapid progression to metastasis, high rate of BRCA1 mutations^4^, chromosome instability^5^ and is more commonly diagnosed in younger women (pre-menopausal) as well as women of African-American descent^6^. It lacks the expression of estrogen and progesterone receptors (ER and PR) as well as the human epidermal growth factor receptor 2 (HER2). Treatment which target these receptors are ineffective in TNBC and the aggressive nature of TNBC drives the urgent need for new treatment targets.

The development and progression of cancer is due to the accumulation of multiple genetic and epigenetic change. DNA methylation is the most well-studied epigenetic change, which culminates in altered gene expression^7^,^8^. DNA methylation is mediated by DNA-methyltransferase (DNMT), which leads to a conformational change of the nucleosomes, where histones are drawn tighter together thereby excluding transcription factors access such that genes cannot be transcribed and expressed. Generally, there is a global decrease of DNA methylation (hypomethylation) in cancer cells, which leads to increased genomic instability^9^. Nevertheless, an increase in DNA methylation (hypermethylation) has been identified at tumour suppressor genes in multiple cancers^10^.

DNA methylation loci are attractive candidates as biomarkers for TNBC as they are more stable than RNA or proteins and are readily detectable in tissue samples and blood^11^. In TNBC, the methylation pattern of a number of cancer-related genes has been analysed^12^. Additionally, methylation patterns have been used to differentiate breast cancer subtypes^13^. Sharma *et al.* discovered that the methylation of the BRCA1 promoter region is associated with worse overall survival and relapse-free survival in TNBC^14^. A recent study by Stirzaker *et al.* used whole genome DNA methylation analysis to identify a signature which divided TNBC into three prognostic subgroups and identified differentially methylated regions (DMRs) associated with overall survival^15^. However, there have been no studies to date that have addressed genome-wide methylation change during disease progression from the primary tumour to lymph node metastasis in TNBC.

In this study, we aimed to identify whether DNA methylation contributed to the altered expression of 38 genes we identified previously in TNBC^16^. By performing whole genome methylation analysis of 23 grade 3 primary invasive ductal carcinomas (IDC) and 11 matched normal adjacent tissues (NAT), we determined that 42% of our TNBC specific genes had significantly altered methylation. Furthermore, by comparing IDC to NAT and IDC to 12 matched lymph node metastases (LN), we identified a set of DNA methylation aberrations associated with the progression of TNBC from primary tumour to LN metastases. We validated the methylation changes of 18 genes associated with LN metastasis with a regional DNA methylation analysis in an independent cohort. Additionally, we identified nine methylation probes, that have significantly altered methylation in LN samples to be associated with survival in TNBC. This is the first whole genome methylation study in TNBC including matched IDC, LN, and NAT samples. We were able correlate the findings of this study with the gene expression results of our previous report using the same sample cohort. A number of previously identified genes show differential methylation suggesting the potential functional relevance of these changes.

## Results

### Methylation profiles are altered in TNBC

We performed 450 K DNA methylation BeadChip array analysis (Illumina) in two independent TNBC cohorts. The study cohort contained 23 TNBC primary IDCs and the validation cohort contained 70 TNBC primary IDCs. All primary tumour samples were compared to three pooled NAT samples and one singular NAT (11 samples in total).

**Figure 1:**
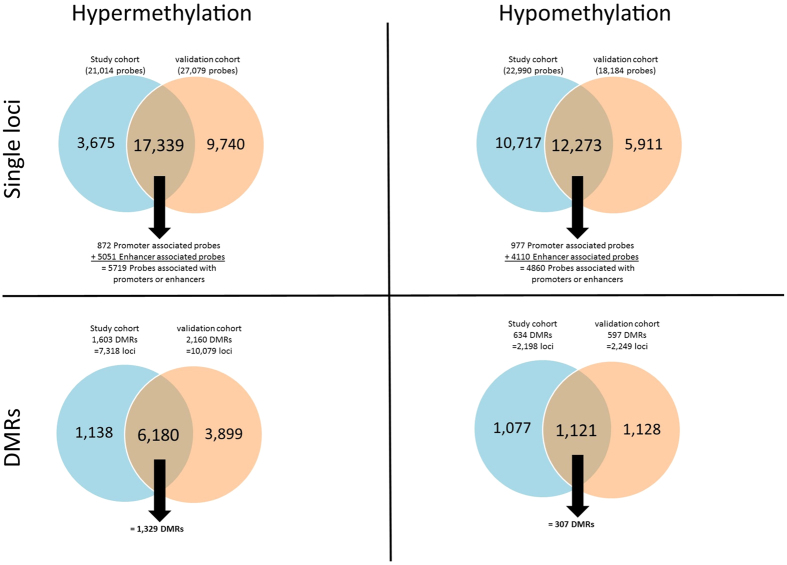
Summary of the DNA methylation comparing primary tumours (IDC) versus matched normal adjacent tissue (NAT) in the study cohort (blue) and the validation cohort (orange). The top two Venn diagrams show hyper- and hypomethylation of single loci, and the bottom two Venn diagrams show hyper- and hypomethylation of differentially methylated regions (DMRs). The number of validated methylation probes is shown in the middle of each Venn diagram. Underneath the validated number of probes, the number of these probes that are located within promoter and enhancer regions is shown (top two Venn diagrams).

By comparing the methylation of the IDC samples to the NAT samples we identified 44,005 differentially methylated probes in the study cohort (52.2% hypomethylated, 47.8% hypermethylated) and 45,263 probes in the validation cohort (40.2% hypomethylated, 59.8% hypermethylated). We validated 29,612 probes (=67.29%) in the independent validation cohort. Of the 29,612 validated probes, 41.4% were hypomethylated and 58.6% were hypermethylated. Within the validated probes there were 1,849 promoter-associated probes (52.8% hypomethylated, 47.2% hypermethylated) and 9,161 probes within enhancer regions (44.9% hypomethylated, 55.1% hypermethylated). In total, there were over 10,000 probes within enhancer or promoter regions that were significantly methylated. Performing a pathway enrichment analysis of these probes identified many important cancer pathways influenced by the respective genes. The pathways with the highest enrichment scores for hypomethylated genes were: Axon guidance, Rap1 signaling pathway, Platelet activation, Mucin type O-Glycan biosynthesis, and MAPK signaling pathway. The pathways with the highest enrichment scores for hypermethylated genes were: ECM-receptor interaction, Pathways in cancer, PI3K-Akt signaling pathway, focal adhesion, and signaling pathways regulating pluripotency of stem cells (Supplementary Table 1). We next identified differentially methylated regions (DMRs, a minimum of three significant consecutive probes). In the study cohort we identified 2,373 DMRs (10,082 probes) and in the validation cohort we identified 2,932 DMRs (12,938 probes), 72.62% (1,756 DMRs/7,523 probes) were common in both cohorts (Supplementary Table 2). The results of this analysis are shown in Fig. 1.

### Genes differentially expressed in TNBC compared to NAT are associated with altered methylation patterns

**Figure 2:**
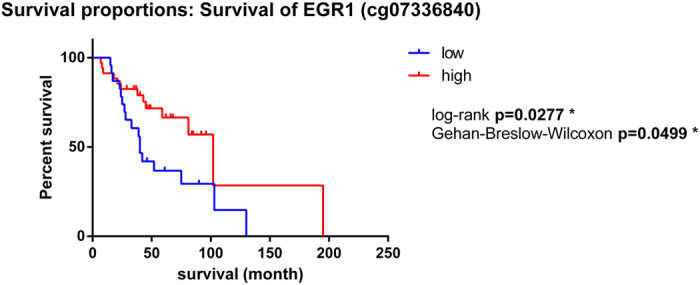
Survival analysis of the *EGR1* probe cg07336840. The y-axis shows the percent of survival of patients within the validation cohort. The x-axis shows the number of months of survival since diagnosis. The blue line represents patients with low DNA methylation of this probe, whereas the red line represents patients with high DNA methylation of this probe.

We have previously identified 66 genes to be differentially expressed in primary tumour samples compared to normal adjacent tissue in TNBC^16^. Therefore, in this study we aimed to identify the contribution of DNA methylation aberration to these gene expression changes. We determined that 26 of the 66 genes had significantly altered methylation of single loci (a total of 63 probes, 47.6% hypomethylated, 52.4% hypermethylated) in both the study and the validation cohorts (Supplementary Table 3). Of the significant probes there were nine within enhancers and four within promoter regions. Additionally, eight of the 66 genes had significantly altered regional methylation (40 probes, 30% hypomethylated, 70% hypermethylated) in both cohorts (Table 1). Of the eight genes with significantly altered regional methylation, one of these (*EGR1*) was significantly associated with overall survival in one probe (cg07336840). High DNA methylation of this probe was associated with better survival as shown in Fig. 2.

### Relationship of methylation changes to altered expression of TNBC-specific genes

Previously we identified 38 genes that had altered expression in the TNBC subtype but not in other breast cancer subtypes, using two independent cohorts^16^. In the first cohort 28 TNBC-specific genes and in the second 14 TNBC specific genes were identified. There were four genes common to both cohorts (*ANKRD30A*, *ANP32E*, *DSC2*, and *IL6ST*). Here, we sought to investigate the DNA methylation changes of these 38 TNBC-specific genes.

**Figure 3:**
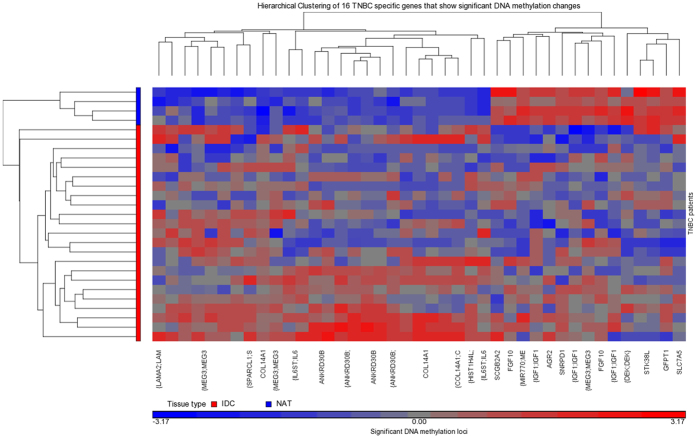
Unsupervised Hierarchical clustering of the DNA methylation of the significant 16/38 TNBC specific genes. Primary tumour TNBC (IDC) samples are shown in red and matched normal adjacent tissue (NAT) samples are shown in blue in the sample tree on the left (y-axis). Genes are clustered along the x-axis. Hypomethylation is shown in blue, hypermethylation is shown in red and equivocal methylation is shown in grey.

We found that 16 of the 38 TNBC-specific genes were associated with differentially methylated probes in the study and validation cohorts (41 probes) (Table 2 and Fig. 3). A set of five genes (*ANKRD30B, COL14A1, IGF1, IL6ST, MEG3*) exhibited regional methylation differences (28 probes) in both cohorts. Three of which showed very strong methylation changes in both cohorts (>20% methylation change), these were *ANKRD30B* (7 hyper-methylated probes), *COL14A1* (6 hyper-methylated probes), and *MEG3* (8 hyper-methylated probes). Of the four TNBC-specific genes that were common in both analyses in our previous study, there was one (*IL6ST*) that showed significantly altered methylation in 3 probes in both cohorts (Table 2). The methylation change for *IL6ST* can be classed as a DMR. However, no probes within these regions were significantly associated with survival (data not shown).

### Methylation changes associated with lymph node metastases

In our previous study^16^ we identified 83 genes that showed altered expression both in primary tumours with LN metastases and in their matched LN metastasis, but were unaltered in lymph node negative tumours. This led to the rationale that the expression of these genes may be affected by DNA methylation and is a contributing factor to tumour progression. To interrogate this, we performed 450 K DNA methylation arrays on 12 LN metastasis samples from the same cohort used in our previous study^16^ and compared the DNA methylation from these samples to that of the NAT samples. A total of 51,563 probes had significantly altered methylation when comparing LN to NAT samples (46.7% hypermethylated, 53.3% hypomethylated) of which, there were 2,350 significant DMRs (these DMRs contain 11,218 probes). Furthermore, 38 of the 83 LN metastasis-associated genes had significantly altered DNA methylation in 107 probes. Of these, 14 genes were present in DMRs (74 probes). The gene expression and DNA methylation changes of these 14 genes is shown in Supplementary Table 4.

**Figure 4:**
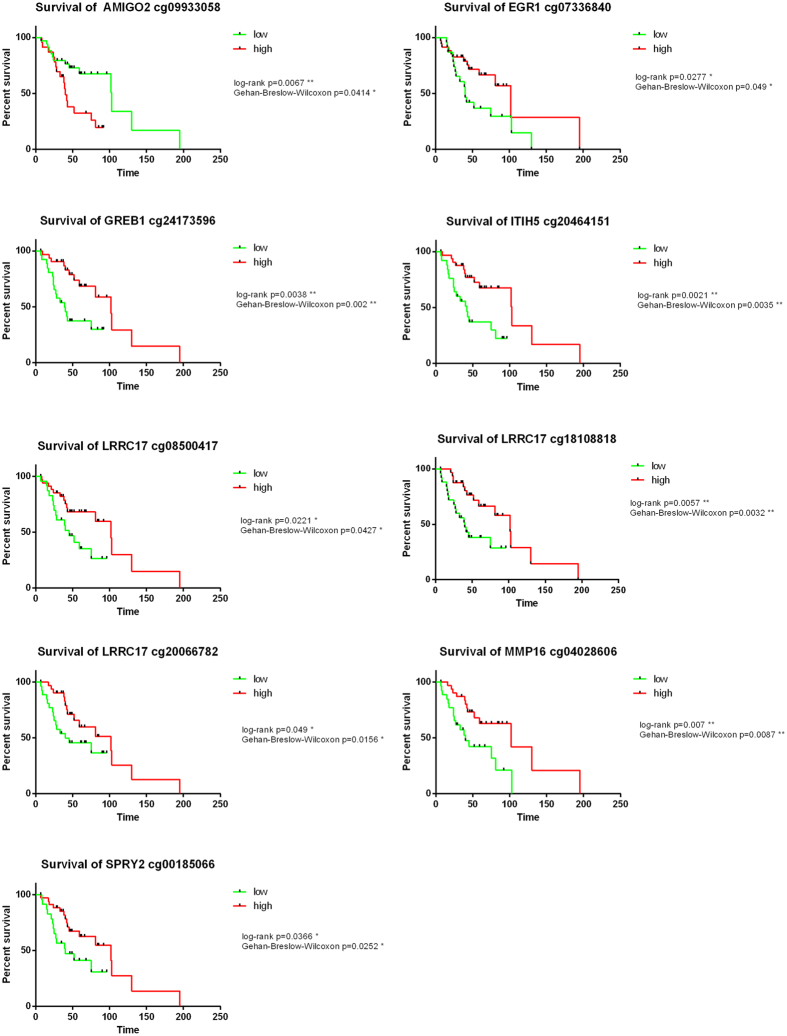
Survival analysis of nine probes that show significant methylation changes comparing lymph node metastasis to matched normal adjacent tissue. The x-axis shows the number of months of survival since diagnosis. The green line represents patients with low DNA methylation of this probe, whereas the red line represents patients with high DNA methylation of this probe.

It was not possible to validate the methylation changes in the validation cohort due to the lack of LN samples. However, we hypothesised that due to their altered expression in LN metastases, they would be associated with survival outcome. We performed survival analysis on the 70 tumour samples from our validation cohort using the methylation analysis of the 74 probes that comprised the 14 DMRs. Nine of the 74 probes were significantly associated with survival in the TNBC validation cohort (cg18108818, cg20464151, cg09933058, cg24173596, cg07336840, cg08500417, cg20066782, cg04028606, cg00185066) (Fig. 4). Eight of these probes (cg18108818, cg20464151, cg24173596, cg07336840, cg08500417, cg20066782, cg04028606, cg00185066) were associated with improved survival when they were highly methylated and one probe (cg09933058) was associated with worse survival when it was highly methylated.

Due to the lack of LN samples in the validation cohort, Methyl-Binding-Domain-Capture (MBDcap) sequencing in an independent cohort of 7 LN and 4 NAT samples was used to validate the direction of DNA methylation of the 38 genes that showed significantly altered methylation when comparing LN to NAT samples in the study cohort. The MBDcap sequencing data analysed 1 kb regions covering all 38 genes associated with LN metastasis (identified in our previous gene expression analyses) starting 2 kb upstream from the first transcription start site to the end of the gene. Of the 38 genes that showed significant altered methylation in 107 probes, 18 of these had significant regional DNA methylation changes in the same direction (hyper/hypomethylation) using MBDcap sequencing data. The results of this analysis can be seen in Table 3.

### Tumour versus lymph node

We have previously reported that miRNA and gene expression patterns are highly similar in LN metastases and the primary tumour within our study cohort^16^,^17^. We next compared whether the methylation changes occurring in LN metastases were similar to those occurring in the primary tumour. The two comparisons of IDC vs NAT and LN vs NAT yielded an overlap of 88.75% (=39,057 probes) or 89.93% (2134 DMRs), indicating that IDC and matched LN metastases DNA methylation alterations are highly similar.

We next compared the DNA methylation of all IDC samples with the DNA methylation of all LN samples to determine methylation changes specifically associated with metastases that were not present in the primary tumour. This comparison revealed that 5,221 probes (58.1% hypomethylated, 41.9% hypermethylated) and 104 DMRs (=366 probes) showed significantly altered methylation. Over 2,000 of these significantly associated probes were located within enhancer or promoter regions. Pathway enrichment analysis revealed the following significant pathways for hypomethylated genes: Inflammatory mediator regulation of TRP channels, Fructose and mannose metabolism, Regulation of lipolysis in adipocytes, Estrogen signaling pathway, and Platelet activation. Pathway enrichment analysis revealed the following significant pathways for hypermethylated genes: Glycosaminoglycan biosynthesis - heparan sulfate/heparin, Axon guidance, Gastric acid secretion, GABAergic synapse, and Glycosaminoglycan biosynthesis - chondroitin sulfate/dermatan sulfate (Supplementary Table 5).

## Discussion

We have previously identified gene expression changes in TNBC primary tumours compared to matched NAT as well as gene expression changes associated with the progression of TNBC from primary tumour to lymph node metastasis. In this study we performed whole genome DNA profiling to determine the contribution of DNA methylation to these gene expression changes as DNA methylation is known to gene silencing events. Here we used the same sample cohort to identify DNA methylation changes that were associated with the previously observed alterations in gene expression by: (1) comparing tumour and matched normal samples and; (2) that were associated with lymph node metastasis.

First, we aimed to identify a global methylation profile of TNBC primary tumour samples (IDC) compared to matched normal adjacent tissue (NAT) to provide information about tumour specific differences. We identified and validated global hypermethylation and hypomethylation that included single loci as well as differentially methylated regions (DMRs) (a minimum of three significant consecutive probes). Since DNA methylation contributes to gene expression changes^18^, the differences in the methylation profiles between these two tissue types were expected. There has only been one other whole genome DNA methylation study in TNBC^15^, which focused on the prognostic value of DNA methylation patterns. The study by Stirzaker *et al.* identified 308 hypermethylated genes by comparing IDC versus NAT samples using Methylation-Binding-Domain Capture sequencing data^15^. We identified 227 (73.7%) of these genes in our study cohort to be significantly hypermethylated. The global analysis of DNA methylation revealed a higher number of hypermethylated probes and DMRs (>17,000 probes, >1,300 DMRs) than hypomethylated probes and DMRs (>12,000 probes, 307 DMRs) comparing the two tissue types, which has also been reported by Stirzaker *et al.*^15^. Interestingly over 10,000 of these probes are located within enhancer or promoter regions. Activation/inactivation of enhancers can affect the transcription of the host gene so that they can act as alternative promoters^19^,^20^,^21^. Our study identified a number of pathways that were associated with altered methylation within enhancer/promoter regions, including the estrogen signalling pathway. In particular, DNA methylation has been associated with hormone receptor status of breast cancer patients^22^,^23^ and our current study suggests that DNA methylation may be involved in the downregulation of the estrogen receptor in TNBC patients, which was also shown in ref. 15.

Next, we focused more specifically on the genes identified in our previous study^16^. There we identified 66 genes to be differentially expressed in tumour versus normal samples. Our current study revealed that 26 of the 66 genes showed significantly altered DNA methylation, which was verified in two independent cohorts. The majority of these (19 genes = 73%) were negatively correlated, such that where gene expression was upregulated, DNA methylation was decreased and vice versa. However, there were genes whose DNA methylation profile was positively correlated with their gene expression (gene expression was upregulated when DNA methylation was increased, and vice versa). This does not mean that the DNA methylation of these genes does not contribute to their gene expression levels as described recently^24^. There Wan *et al.*^24^ hypothesised two mechanisms of DNA methylation-dependent gene regulation: (1) the dogma of gene repression due to DNA methylation; and (2) gene activation through DNA methylation. They found positively correlated gene-methylation relationships to be in more conserved regions and mainly in promoter regions, which suggests that these positive correlations have a regulatory role and do not just happen by chance. It may also indicate differential promoter usage as seen in ref. 25.

Our previous study found that *EGR1* gene expression is downregulated comparing tumour versus normal. The methylation of *EGR1* was found to be negatively correlated with its gene expression in our TNBC cohort, where there were five significant CpG methylation probes that were hypermethylated in both cohorts comparing these two tissue types. This gene has also been identified by other studies comparing IDC versus NAT in TNBC^26^. It is a zinc finger protein that acts as a transcriptional regulator functioning as a tumour suppressor by regulating other tumour suppressors including *TGFβ1*, *PTEN*, *p53*, and fibronectin^27^. We were able to correlate the DNA methylation of one of the significant probes within *EGR1* (cg07336840) with overall survival (p < 0.05). High DNA methylation at this locus is significantly associated with better overall survival. Interestingly, the study by Stirzaker *et al.* identified a DMR located in the *WT-1* gene to be associated with poorer overall survival in TNBC patients^15^. *EGR1* and *WT-1* are members of the same family (early growth response – zinc-finger family) but with mostly opposing functions, *EGR1* activates the transcription of genes that *WT-1* represses^28^,^29^.

We previously identified that the gene expression of 38 genes is specific to TNBC^16^. Here we investigated the DNA methylation levels of these genes to determine the contribution of DNA methylation to gene expression. We showed that half (16/38) of the TNBC specific genes showed significantly altered DNA methylation at 41 probes; and of these there are five genes classed as DMRs (28 probes). These five genes are *ANKRD30B, COL14A1, IGF1, IL6ST,* and *MEG3. ANKRD30B* has been shown to be expressed in breast, brain, and testicular tumours^30^ but it has not been studied in TNBC to date. Stirzaker *et al.*^15^ also identified significant hypermethylation of *COL14A1* when comparing tumour versus normal tissue^15^. However, there is a need for functional analysis of *COL14A1* in TNBC. *IGF1* has been the focus of multiple TNBC studies and is known to regulate cell proliferation and survival, and has been suggested as a potential treatment target for TNBC^31^,^32^,^33^. We have previously identified *IL6ST* as a TNBC-specific gene and its gene expression to be associated with overall survival (increased gene expression → better survival)^16^. Our previous study showed *MEG3* is associated with lymph node metastasis^16^. It is a long non-coding RNA that is known to be down-regulated in multiple cancers and to regulate cell proliferation through the p53-tumour suppressor pathway^34^,^35^.

Due to its aggressive nature and increased number of metastasis, TNBC patients have much poorer outcomes relative to other subtypes. Therefore, we aimed to identify differences during early cancer progression from the primary tumour site to lymph node metastasis (LN). Eighty three genes were previously shown to be associated with LN metastasis^16^. Here we revealed that the expression of 38 of these genes may be influenced by methylation changes at single DNA methylation loci. Of these there are 14 genes that were differentially methylated in DMRs (over 74 probes). The survival analysis on our validation cohort using these 74 probes (Fig. 4) showed that nine probes were significantly associated with survival. Seven probes associated with five genes (*SPRY2, EGR1, GREB1, ITIH5, LRRC17*) were associated with better survival having higher methylation, whereas one probe for *AMIGO2* (cg09933058) shows better survival with low methylation. Interestingly, of the significant DMRs comparing IDC versus NAT only one probe showed significant association with survival (*EGR1* (cg07336840)) which is also in a DMR comparing LN versus NAT. *SPRY2* and *AMIGO2* have not previously been studied in TNBC. However, *SPRY2* is a known tumour suppressor that regulates the RAS-ERK pathway^36^,^37^. *AMIGO2* has been shown to be differentially expressed in other cancers including gastric adenocarcinomas, and it is known to effect ploidy, chromosomal stability, cell adhesion/migration, and tumourigenicity^38^. It also controls cell survival and angiogenesis via Akt activation^39^. The DMR of *LRRC17* is associated with survival in three probes (high methylation/better survival). Interestingly, this gene has been identified as a TNBC specific gene previously^40^. However, no functional analysis of *LRRC17* in TNBC has been done. Promoter methylation of the tumour suppressor *ITIH5* has been suggested as early breast cancer detection biomarker^41^. Finally, *GREB1* is a key estrogen regulator^42^ and is expressed in hormone responsive breast cancers^43^ but not in TNBC^16^.

Due to the lack of LN samples in our validation cohort, we utilised Methylation-Binding-Domain Capture sequencing (MBDcap seq) data from 7 LN and 4 NAT samples to validate the direction of methylation change of the genes that are associated with LN metastasis. Differential methylation was validated for 18 of the 38 genes associated with LN metastasis. The MBDcap seq provided regional methylation analysis covering 2 kb upstream from the first transcription start site to the end of the gene of interest, these regions were broken up into 1 kb tiles. The majority of genes (12 of 14) show a negative correlation between gene expression and DNA methylation. However, there are four genes that show negative and positive correlation in different probes. These are *TSHZ2* (known to be down-regulated in breast and prostate cancer^44^)*, ITIH5* (promoter methylation is an early breast cancer detection biomarker^41^)*, GREB1* (estrogen regulator^42^,^43^), *MEG3* (long non-coding RNA known to be down-regulated in cancer^34^,^35^), and *RELN* (methylated and down-regulated in pancreatic cancer, where its expression has been associated with increased cell motility, invasiveness and colony-forming ability^45^ but has not been described in breast cancer). This could mean that some loci overpower others or potentially, that during tumour progression to lymph nodes, the methylation becomes tissue specific and changes. Further research into loci specific DNA methylation during cancer progression is urgently needed to explain these phenomena. Additionally, we reviewed the connection of the 18 validated genes to epithelial-mesenchymal transition (EMT) (a process which cells undergo to travel to distant sites, leading to distant cancerous disease). The majority of these genes (12/18) have a known connection to EMT, which we summarised in Table 4. These findings support the importance of these genes during cancer progression.

In conclusion, this is the first whole genome DNA methylation analysis in a TNBC cohort including matched lymph node metastases. Here we identified and validated a global DNA methylation profile, which we correlated with our previously published gene expression findings in tumour, matched lymph node and matched normal adjacent tissue. Our findings show that DNA methylation contributes to the deregulation of gene expression changes and is associated with overall survival.

## Methods

### Study design

The study cohort comprised a total of 23 grade three invasive ductal carcinomas (IDC), 12 matched lymph node metastasis (LN), and 11 matched normal adjacent tissues (NAT), from which DNA was isolated and screened using the Illumina HumanMethylation450 BeadChips to reveal DNA methylation changes across the genome. All samples in the study cohort were obtained as formalin-fixed, paraffin-embedded (FFPE) blocks from the archives of the Hunter Area Pathology Service, John Hunter Hospital, Newcastle, Australia. This cohort has been described previously^17^. A pathologist confirmed the triple negative phenotype, areas of NAT, invasive cancer and LN metastasis. As previously described, 1.5 mm punch biopsies were used to isolate areas of IDC, LN and NAT from these sections.

The validation cohort used in this study contained 70 IDC samples derived from the Australian Breast Cancer Tissue Bank (ABCTB) and the same 11 NAT samples used in the study cohort. All participants consented to the use of their tissue in this study. Details regarding this cohort are shown in Table 5.

To assess the direction of DNA methylation in LN metastasis samples, we utilised Methylation-Binding-Domain-Capture sequencing data from 7 LN samples and 4 NAT samples (Supplementary Table 6).

### Ethics statement

All experiments were performed in accordance with approved guidelines and regulations.

This study, including all experimental protocols, was granted a waiver of consent in accordance with the *National Statement on Ethical Conduct in Research Involving Humans*.

This study, including all experimental protocols, complies with the Helsinki Declaration with ethical approval from the Hunter New England Human Research Ethics Committee (Approval number: 09/05/20/5.02).

Written informed consent was obtained from all patients included in this study.

### DNA extraction

The Gentra Puregene Tissue Kit (Qiagen, Venlo, Limburg, Netherlands) was used to isolate DNA from FFPE tissue following the manufacturers’ instruction with few alterations. The protocol including the alterations was as follows.

All biopsy samples were placed in 1.5 ml tubes. Five hundred microliters of Xylene were added in a fume hood and incubated with constant gentle mixing at 55 °C. This was followed by centrifugation at 16,000 g for 3 minutes. The supernatant was discarded and the Xylene wash step repeated twice. Five hundred microliters of 100% ethanol was added and incubated for 5 mins under constant mixing at room temperature. For the cell lysis, 300 μl of Cell Lysis Solution was added to each tube and incubated for 10 mins at 70 °C. Twenty microliters Proteinase K (20 mg/ml) were added to each sample, mixed for 20 sec and incubated at 55 °C overnight. On day two, a further 10 μl Proteinase K was added to each sample, mixed and again incubated overnight at 55 °C. On day three, 5 freeze-thaw cycles were performed, 5 mins on dry ice and 5 mins at 95 °C. All samples were brought back to room temperature. Two microlitres of RNase A solution (4 mg/ml) was added to each sample, inverted 25 times and then incubated at 37 °C for one hour. This was followed by a protein precipitation step by adding 100 μl Protein Precipitation solution to the cell lysates, mixed, incubated on ice for 5 mins, followed by centrifugation for 5 mins at 4 °C at × 21,100 g (full speed). For DNA precipitation the supernatant was transferred into a new tube and 300 μl of 100% isopropanol were added. The solutions were mixed by inverting 30 times. Followed by full speed centrifugation at 4 °C for 15 mins. Supernatant was discarded, and 300 μl cold 70% ethanol were added. All samples were centrifuged for one minute at full speed, the supernatant discarded and all samples were subject to further centrifugation for a further minute at full speed. The DNA pellet was air-dried and dissolved in 20 μl of DNAse-free water with constant mixing for one hour. The DNA was stored at −20 °C until used.

DNA was quantitated using the Qubit dsDNA BR Assay Kit according to the manufacturer’s instructions (Life Technologies, Carlsbad, CA, United States of America).

### Illumina Infinium HD FFPPE methylation arrays

The array results have been deposited in Gene Expression Omnibus (GEO) with Accession No. GSE78758.

The Infinium HD FFPE quality control (QC) Assay (Illumina, San Diega, CA, United States of America) was used to assess the integrity of the DNA used for methylation analysis. It was performed using a Real-time PCR assay according to the manufacturers’ instructions (Applied Biosystems 7500 Fast Real-Time PCR system). All samples were assayed in triplicates. Two microliters of genomic DNA (at 1 ng/μl) was used for each reaction. The threshold cycle (Ct) was calculated for each individual sample. Replicates where the Ct diverged by more than half a unit were excluded. The average Ct was calculated for each sample as well as the QC template reagent (Illumina). To calculate the delta Ct (ΔCt) the average Ct of the QC template reagent was subtracted from the average Ct of each sample. The ΔCt had to remain below 5 for the samples to pass the quality control test. Of the initial 37 IDC samples, 27 had enough DNA (>250 ng) for further analysis, and 23 of these passed the quality control (ΔCt < 5), all 12 LN samples passed the QC stage as well as the 3 NAT pooled samples and one of the single NAT samples passed the QC.

Bisulfite conversion was performed using the EZ-96 DNA Methylation Kit (Zymo Research, Irvine, CA, United States of America).

Next, FFPE Restoration was undertaken following the Infinium HD FFPE Restore Protocol (Illumina).

Infinium HD FFPE Methylation Assay (Illumina) with the hybridisation, washing and staining of the arrays as well as the scanning (iScan) of the HumanMethylation 450 K BeadChip arrays was performed using the manufacturers’ instructions.

### Methylation array analysis

The data from all samples was imported in form of the idat files into Genomic Suite 6.6 (Partek, St Louis, Missouri, United States of America) and Illumina normalisation was performed. ANOVA analysis was performed to detect differentially methylated probes between groups (IDC versus NAT, LN versus NAT, and IDC versus LN). Significance was granted if p < 0.05 and the estimated difference between groups (Δβ) was <−0.1 or >0.1, signifying a methylation change of at least 10%.

These analyses were performed on single loci (=probes on the BeadChips) and on differentially methylated regions (DMR) – a minimum of three significant consecutive probes. The focus in this study was the 38 triple negative specific genes which we identified in our previous study^16^.

All samples of the validation cohort were treated and analysed in the same way. Due to the lack of LN samples in this cohort, only the IDC versus NAT comparisons were performed.

Pathway enrichment analysis was performed using Genomic Suite 6.6 (Partek). All significant probes were filtered to include probes that are within enhancer and/or promoter regions. These were then used for pathway enrichment analysis, which is a tool within Genomic Suite 6.6 (Partek). The enrichment score is the negative natural log of the enrichment p-value derived from the Fisher’s exact test of the pathway enrichment analysis.

### MDB-Cap Sequencing

We compared our findings (comparing LN versus NAT) with MBDcap sequencing data provided by Dr. Clare Stirzaker and Prof. Susan Clark from the Garvan Institute Sydney. The methylation profiling was performed as previously described in ref. 15. This analysis provides regional DNA methylation information therefore a strict validation with our CpG-specific analysis was not possible. The regions that were analysed using the MBDcap data included our genes of interest (83 genes that are associated with LN metastasis, identified in ref. 16) starting 2 kb upstream of the first transcription start site (TSS) to the 3′ end of the genes. These regions are broken up into 1kb tiles. The methylation profiling was performed as described by Stirzaker, *et al.*^15^ where the same sample cohort had been used (excluding the LN samples) and methylation differences between LN and NAT samples were assessed using a Student’s t-test (using the R base package) (p < 0.05).

## Additional Information

**How to cite this article**: Mathe, A. *et al.* DNA methylation profile of triple negative breast cancer-specific genes comparing lymph node positive patients to lymph node negative patients. *Sci. Rep.* **6**, 33435; doi: 10.1038/srep33435 (2016).

## Supplementary Material

Supplementary Table 1

Supplementary Table 2

Supplementary Table 3

Supplementary Table 4

Supplementary Table 5

Supplementary Table 6

## Figures and Tables

**Table 1:** DNA methylation of 8 validated DMRs comparing IDC vs NAT.

DMR #	Column ID	CHR	UCSC_REFGENE_GROUP	ENHANCER (E)/PROMOTER(P)	RELATION_TO_UCSC_CPG_ISLAND[1]	UCSC_REFGENE_NAME			DNA methylation			
							Gene expression		Study cohort		Validation cohort	
							Fold change	p-value	p-value (IDC vs. NAT)	Methylation Difference (IDC vs. NAT)[2]	p-value (IDC vs. NAT)	Methylation difference (IDC vs. NAT)
1	cg25824760	18	TSS200		Island	ANKRD30B	−11.36	1.11E–17	0.003	0.127	0.02	0.121
	cg23703062	18	TSS200		Island				0.035	0.183	0.027	0.191
	cg13266435	18	TSS200		Island				0.008	0.237	0.037	0.179
	cg21293934	18	TSS200		Island				0.017	0.21	0.03	0.168
	cg24061208	18	1st Exon, 5′UTR		S_Shore				0.004	0.299	0.017	0.271
	cg03014326	18	1st Exon, 5′UTR		S_Shore				0	0.441	0.003	0.431
	cg21281009	18	1st Exon, 5′UTR		S_Shore				0.013	0.206	0.045	0.136
2	cg23196831	8	1st Exon, 5′UTR		Island	COL14A1	−5.57	4.10E–14	0.049	0.229	0.034	0.261
	cg26626663	8	5′UTR		S_Shore				0.015	0.366	0.011	0.349
	cg12065840	8	5′UTR		S_Shore				0.032	0.284	0.085	0.223
	cg23586322	8	5′UTR		S_Shore				0.014	0.36	0.04	0.276
	cg23281803	8	5′UTR		S_Shore				0.014	0.303	0.004	0.296
	cg25448355	8	gene body	**E**					0	0.189	0	0.204
3	cg09102257	5	gene body		Island	EGR1	−12.87	8.24E–38	0	0.514	0	0.508
	cg07336840	5	gene body		Island				0	0.329	0	0.311
	cg23029363	5	gene body		Island				0	0.267	0	0.257
	cg19729803	5	gene body		S_Shore				0	0.283	0	0.291
	cg01107476	5	3′UTR		S_Shore				0.002	0.238	0.005	0.232
4	cg23046919	12	3′UTR			IGF1	−5.83	1.42E–18	0.02	−0.163	0.008	−0.152
	cg12742178	12	gene body	**E**					0.017	−0.216	0.035	−0.178
	cg00264799	12	gene body	**E**					0.038	−0.116	0.011	−0.135
5	cg24729879	5	5′UTR		N_Shore	IL6ST	−3.20	1.92E–23	0.001	0.225	0.001	0.218
	cg16375820	5	5′UTR		N_Shore				0.002	0.167	0.004	0.142
	cg15219433	5	TSS200	**P**	Island				0.01	0.122	0.004	0.144
6	cg12922492	7	gene body	**E**		INHBA	5.23	3.52E–60	0.024	−0.2	0.025	−0.184
	cg01412469	7	TSS1500						0.007	−0.13	0.011	−0.136
	cg15291905	7	5′UTR	**E**	N_Shelf				0.015	−0.195	0.001	−0.252
7	cg14119337	14	TSS1500		N_Shore	MEG3	−3.39	3.52E–68	0.003	0.179	0.006	0.14
	cg12967319	14	TSS1500		N_Shore				0.026	0.137	0.012	0.15
	cg04304932	14	TSS1500		Island				0.002	0.203	0.031	0.127
	cg15419911	14	gene body		Island				0.004	0.195	0.017	0.177
	cg14123427	14	gene body		Island				0.001	0.21	0.012	0.183
	cg26374305	14	gene body		Island				0	0.337	0	0.329
	cg08698721	14	gene body		Island				0	0.19	0.006	0.168
	cg24183098	14	gene body		S_Shore				0.039	−0.143	0.034	−0.151
	cg03039990	14	TSS1500						0.049	−0.151	0.006	−0.23
8	cg04212239	3	gene body	**P**	S_Shore	SMC4	3.15	1.08E–37	0	−0.178	0	−0.155
	cg13783238	3	TSS1500		S_Shelf				0.015	−0.213	0.009	−0.212
	cg12785694	3	TSS1500		S_Shelf				0.003	−0.28	0.001	−0.329
	cg09100593	3	TSS200		S_Shelf				0.001	−0.3	0	−0.312

These 8 DMRs are within 8 of 66 validated genes from previous study.

[1] Every CpG island consist of N- and S Shores next to it, which are neighboured by N- and S-shelfs. First N-shelf, N-shore, CpG island, S-shore, and last S-shelf

[2] Methylation difference between IDC and NAT shows % of methylation change/100.

**Table 2:** Validated DNA methylation of 41 probes that are significantly different in the comparison of IDC vs NAT.

Column ID	CHR	UCSC_REFGENE_GROUP	ENHANCER(E)/PROMOTER(P)	RELATION_TO_UCSC_CPG_ISLAND[1]	UCSC_REFGENE_NAME			DNA methylation			
						Gene expression		Study cohort		Validation cohort	
								p-value	Fold change	p-value (IDC vs. NAT)	Methylation Difference (IDC vs. NAT)[2]	p-value (IDC vs. NAT)	Methylation difference (IDC vs. NAT)
cg12565635	7	TSS1500			AGR2	8.72E-12	−3.01	0.038	−0.137	0.003	−0.161
cg25824760	18	TSS200		Island	ANKRD30B	0.00046	−2.24	0.003	0.127	0.02	0.121
cg23703062	18	TSS200		Island				0.035	0.183	0.027	0.191
cg13266435	18	TSS200		Island				0.008	0.237	0.037	0.179
cg21293934	18	TSS200		Island				0.017	0.21	0.03	0.168
cg24061208	18	1st Exon, 5′UTR		S_Shore				0.004	0.299	0.017	0.271
cg03014326	18	1st Exon, 5′UTR		S_Shore				0	0.441	0.003	0.431
cg21281009	18	1st Exon, 5′UTR		S_Shore				0.013	0.206	0.045	0.136
cg23196831	8	1st Exon, 5′UTR		Island	COL14A1	4.9E-09	−3.43	0.049	0.229	0.034	0.261
cg26626663	8	5′UTR		S_Shore				0.015	0.366	0.011	0.349
cg12065840	8	5′UTR		S_Shore				0.032	0.284	0.085	0.223
cg23586322	8	5′UTR		S_Shore				0.014	0.36	0.04	0.276
cg23281803	8	5′UTR		S_Shore				0.014	0.303	0.004	0.296
cg25448355	8	gene body	**E**					0	0.189	0	0.204
cg04239786	5	gene body	**E**		FGF10	7.7E-11	−2.34	0.038	−0.126	0.02	−0.138
cg16204420	5	gene body						0.001	−0.231	0.001	−0.22
cg20041567	6	1st Exon, TSS1500		Island	HIST1H4L	0.012	2.174	0.012	0.17	0.002	0.219
cg23046919	12	3′UTR			IGF1	1.21E-08	−2.96	0.02	−0.163	0.008	−0.152
cg12742178	12	gene body	**E**					0.017	−0.216	0.035	−0.178
cg00264799	12	gene body	**E**					0.038	−0.116	0.011	−0.135
cg24729879	5	5′UTR		N_Shore	IL6ST	7.53E-06	−2.36	0.001	0.225	0.001	0.218
cg16375820	5	5′UTR		N_Shore				0.002	0.167	0.004	0.142
cg15219433	5	TSS200	**P**	Island				0.01	0.122	0.004	0.144
cg23143313	6	gene body		Island	LAMA2	7.43E-07	−2.02	0.019	0.136	0.005	0.161
cg08884591	11	TSS1500			SCGB2A2	4.01E-05	−5.05	0.008	−0.292	0.003	−0.301
cg06372223	16	gene body		N_Shelf	SLC7A5	0.002	2.09	0.007	−0.197	0.001	−0.245
cg08003102	4	TSS1500			SPARCL1	9.09E-06	−2.65	0.009	0.194	0.034	0.141
cg14119337	14	TSS1500		N_Shore	MEG3	3.52E-68	−3.39	0.003	0.179	0.006	0.14
cg12967319	14	TSS1500		N_Shore				0.026	0.137	0.012	0.15
cg04304932	14	TSS1500		Island				0.002	0.203	0.031	0.127
cg15419911	14	gene body		Island				0.004	0.195	0.017	0.177
cg14123427	14	gene body		Island				0.001	0.21	0.012	0.183
cg26374305	14	gene body		Island				0	0.337	0	0.329
cg08698721	14	gene body		Island				0	0.19	0.006	0.168
cg24183098	14	gene body		S_Shore				0.039	−0.143	0.034	−0.151
cg03039990	14	TSS1500						0.049	−0.151	0.006	−0.23
cg08451113	6	gene body		N_Shore	DEK	0.0003	2.01	0.007	−0.118	0.007	−0.133
cg06845268	2	TSS1500		S_Shore	GFPT1	0.0002	2.03	0.028	−0.187	0.007	−0.242
cg26212229	18	TSS1500		N_Shore	SNRPD1	0.0001	2.06	0.026	−0.18	0.011	−0.183
cg23433370	12	TSS200	**P**	N_Shore	STK38L	3.86E-	2.25	0	−0.336	0	−0.35
cg07240557	12	TSS200	**P**	N_Shore				0	−0.215	0	−0.212

These are associated with 16 of the 38 TNBC specific genes identified in our previous study

[1] Every CpG island consist of N- and S Shores next to it, which are neighboured by N- and S-shelfs. First N-shelf, N-shore, CpG island, S-shore, and last S-shelf.

[2] Methylation difference between IDC and NAT shows % of methylation change/100.

**Table 3:** Validation of the direction of methylation comparing lymph node metastasis (LN) to matched normal adjacent tissue (NAT).

Gene name	LNvNAT									
	450K array					MBDcap seq			Gene expression	
	p-value	Methylation difference	Mapinfo	ENHANCER(E)/PROMOTER(P)	UCSC_REFGENE_GROUP	p-value	Fold change	Region	Fold change	p-value
GREB1	0.007	−0.207	11673928	TSS1500	0.045	−2.329	Chr2: 11722242-11723241	−1.57	0.000129	
	0.015	−0.160	11674057	TSS200						
	0.008	−0.180	11674557	5′UTR						
	0.048	−0.234	11724901	gene body						
	0.005	−0.173	11734257	gene body						
	0.040	−0.132	11761803	gene body						
RBMS3	0.003	−0.176	29782270	**E**	gene body	0.032	−2.538	chr3: 29345803-29346802	−2.3	1.10E-05
						0.049	−2.270	chr3: 29404803-29405802		
						0.009	−3.316	chr3: 29434803-29435802		
						0.010	−3.257	chr3: 29481803-29482802		
						0.049	−2.277	chr3: 29488803-29489802		
						0.009	−3.290	chr3: 29498803-29499802		
						0.010	−3.263	chr3: 29516803-29517802		
						0.040	−2.393	chr3: 29630803-29631802		
						0.021	−2.795	chr3: 29636803-29637802		
						0.040	−2.393	chr3: 29665803-29666802		
						0.011	−3.191	chr3: 29748803-29749802		
						0.021	−2.796	chr3: 29843803-29844802		
						0.040	−2.393	chr3: 29871803-29872802		
						0.003	−4.143	chr3: 29921803-29922802		
						0.005	−3.667	chr3: 29962803-29963802		
MME	0.023	−0.104	154900894		3′UTR	0.001	−5.259	chr3: 154794913-154795912	−3.06	3.65E-07
PLSCR4	0.000	0.141	145968692		5′UTR	0.007	3.496	chr3: 145941967-145942966	−2.3	1.10E-05
IGSF10	0.001	−0.101	151176684		TSS200	0.041	−2.379	chr3: 151172498-151173497	−2.26	4.52E-05
APOD	0.017	−0.135	195299575	**E**	gene body	0.011	−3.205	chr3: 195296077-195297076	−9.46	2.33E-0.6
LIFR	0.008	−0.140	38535574	**E**	5′UTR	0.048	−2.284	chr5: 38484508-38485507	−2.43	5.93E-05
						0.025	−2.678	chr5: 38522508-38523507		
						0.040	−2.393	chr5: 38524508-38525507		
	0.014	−0.284	38595383	1st Exon 5′UTR	0.020	−2.813	chr5: 38562508-38563507			
	0.045	−2.323	chr5: 38575508-38576507							
IL20RA	0.002	−0.193	137362747		gene body	0.025	−2.697	chr6: 137323299-137324298	−1.7	1.49E-05
						0.008	−3.424	chr6: 137348299-137349298		
CD36	0.029	−0.193	80252533	**E**	5′UTR	0.040	−2.393	chr7: 80009891-80010890	−2.57	2.77E-06
	0.032	−0.137	80274687		TSS1500	0.040	−2.393	chr7: 80254891-80255890		
						0.040	−2.393	chr7: 80285891-80286890		
LRRC17	0.004	0.150	102574445	**E**	gene body	0.047	2.303	chr7: 102561344-102562343	−1.67	6.38E-05
	0.012	0.112	102574475	**E**	gene body					
	0.037	0.110	102574504	**E**	gene body					
	0.039	0.169	102583144		gene body					
AGR3	0.000	−0.139	16922492		TSS1500	0.025	−2.697	chr7: 16898614-16899613	−2.87	1.44E-08
RELN	0.010	−0.148	103279088	**E**	gene body	0.013	−3.083	chr7: 103216964-103217963	−1.8	0.000112
						0.040	−2.393	chr7: 103220964-103221963		
						0.040	−2.393	chr7: 103250964-103251963		
						0.008	−3.403	chr7: 103261964-103262963		
	0.039	−0.213	103301932	**E**	gene body	0.037	−2.449	chr7:103348964-103349963		
						0.006	−3.547	chr7:103404964-103405963		
						0.029	−2.595	chr7:103449964-103450963		
						0.034	−2.495	chr7:103450964-103451963		
						0.037	−2.451	chr7:103566964-103567963		
CDCA2	0.017	−0.204	25317638		5′UTR	0.044	−2.345	chr8:25320513-25321512	1.9	0.000302
						0.005	−3.704	chr8:25321513-25322512		
						0.006	−3.610	chr8:25325513-25326512		
MMP16	0.049	−0.144	89053427	**E**	3′UTR	0.010	−3.247	chr8:89313718-89314717	−1.57	9.02E-05
	0.027	−0.222	890966							
	0.041	−0.101	89218697	**E**	gene body					
	0.036	−0.113	89231006	**E**	gene body					
	0.042	−0.198	89241632	**E**	gene body					
	0.039	−0.194	89255591	**E**	gene body					
SVEP1	0.005	0.243	113308063	**E**	gene body	0.005	−3.650	chr9:113134161-113135160	−1.87	1.43E-05
						0.001	−4.528	chr9:113173161-113174160		
						0.001	4.632	chr9:113179161-113180160		
						0.033	−2.510	chr9:113201161-113202160		
	0.008	−0.122	113316190	**E**	gene body	0.012	−3.147	chr9:113228161-113229160		
						0.020	2.810	chr9:113266161−113267160		
						0.005	−3.681	chr9:113268161-113269160		
						0.001	−5.016	chr9:113324161-113325160		
ITIH5	0.045	−0.147	7614151		gene body	0.032	−2.539	chr10:7628962-7629961	−1.78	3.16E-05
	0.000	−0.241	7614570		gene body	0.029	−2.592	chr10:7644962-7645961		
	0.042	−0.171	7617637		gene body	0.025	−2.680	chr10:7646962-7647961		
	0.048	−0.142	7618474		gene body	0.045	−2.329	chr10:7649962-7650961		
	0.028	−0.174	76514031	**E**	gene body	0.031	−2.548	chr10:7662962-7663961		
	0.022	−0.101	7661622		TSS200	0.001	−4.689	chr10:7699962-7700961		
	0.027	−0.115	7662761		TSS1500					
MEG3	0.003	−0.120	101291100		TSS1500	0.005	−3.724	chr14:101318445-101319444	−2.8	1.79E-08
	0.033	−0.201	101296297		gene body	0.026	−2.672	chr14:101319445-101320444		
	0.020	−0.223	101317622		TSS1500					
TSHZ2	0.019	0.239	51592177		gene body	0.048	−2.287	chr20:51601946-51602945	−2.33	3.94E-05
						0.005	−3.707	chr20:51684946-51685945		
						0.001	−4.547	chr20:51752946-51753945		
						0.000	−6.638	chr20:51790946-51791945		
						0.038	−2.422	chr20:51819946-51820945		
	0.047	−0.190	52029943		3′UTR	0.041	−2.386	chr20:51825946-51826945		
						0.035	−2.488	chr20:51879946-51880945		
						0.026	−2.672	chr20:51914946-51915945		
						0.043	−2.353	chr20:51928946-51929945		
						0.037	2.453	chr20:51956946-51957945		
	0.041	−01.19	52036204		3′UTR	0.021	−2.803	chr20:51995946-51996945		
						0.036	−2.457	chr20:52006946-52007945		
						0.050	−2.268	chr20:52049946-52050945		
						0.016	2.954	chr20:52082946-52083945		
						0.046	−2.311	chr20:52103946-52104945		

Genes in the first column were identified to be associated with LM in our previous study. Using the 450K methylation arrays we identified single loci within these genes to be differentially methylated comparing LN versus NAT samples (mapinfo shows location of the significant loci). The Methylation-Binding-Domain-Capture sequencing (MBDcap seq) provides regional methylation analysis. The analysed regions start 2kb upstream from the first transcription start side to the end of the gene in 1kb tiles.

**Table 4:** Connection of 18 validated genes, which are associated with lymph node metastasis, to epithelial-mesenchymal transition (EMT).

Gene	Connection to EMT	Ref.
GREB1	EMT is regulated by 17β-estradiol which can be regulated by GREB1	46
RBSM3	is up-regulated during TGF-ß1-induced EMT. No direct connection to EMT in TNBC has been shown	47
MME	there is no known connection to EMT	
PLSCR4	there is no known connection to EMT	
IGSF10	there is no known connection to EMT	
LIFR	is targeted by miR-9 (EMT related microRNA), which is upregulated in breast cancer. LIFR acts as metastasis supressor	48
IL20RA	there is no known connection to EMT	
CD36	high level of CD36 (fatty acid transporter) promote EMT	49
LRRC17	there is no known connection to EMT	
AGR3	is regulated by ZEB1 (key regulator of EMT)	50
RELN	interacts with SNAIL (key regulator of EMT)	51
CDCA2	its gene expression can predict metastasis outcome in synovial sarcomas	52
MMP16	is well known to play a role in EMT; high expression leads to tumour invasion; is targeted by miR-200 (key microRNA in EMT)	53
SVEP1	is activated by TNFα (a pro-inflammatory cytokine able to affect adhesion and migration, and to induce EMT)	54
ITIH5	inactivation of ITIH5 promotes cancer progression in bladder cancer; no direct connection to EMT in TNBC to date	55
MEG3	known regulator of EMT, targets TGF-ß receptor genes (EMT regulators); inhibits cell proliferation and induces apoptosis (through p53)	48
TSHZ2	down-regulation of TSHZ2 releases GLI1 (which can induce EMT)	56

**Table 5:** Sample information from the validation cohort.

Patient Number	Tumour grade	Age at diagnosis	Month of follow up/to death	Follow up Status
1	1	51.94	186	Died from other cause
2	3	52.1	44	Died from disease
3	3	37	16	Died from disease
4	3	66	82	Died from disease
5	3	76	113	Alive no disease
6	3	45	40	Died from disease
7	3	71	68	Alive no disease
8	3	62	52	Alive no disease
9	3	81	8	Died from other cause
10	3	75	17	NA
11	3	45	35	Died from other cause
12	3	42	140	Alive no disease
13	3	41	39	Died from disease
14	3	37	172	Alive no disease
15	3	52.55	61	Died from disease
16	3	68.26	NA	Died from other cause
17	3	70	33	Died from disease
18	3	37	7	Alive no disease
19	3	71	17	Died from disease
20	3	74	170	Alive no disease
21	3	41	23	Died from disease
22	3	59	66	Alive no disease
23	3	59	21	Died from disease
24	3	83	46	Alive no disease
25	3	42	37	Died from other cause
26	3	32	66	Alive no disease
27	3	44	29	Died from other cause
28	3	35	46	Alive no disease
29	3	43	62	Died from other cause
30	3	61	48	Alive no disease
31	3	39	40	Died from other cause
32	2	55.46	188	Died from other cause
33	2	29.09	NA	Died from disease
34	3	32	49	Alive no disease
35	3	74	24	Died from disease
36	3	45	169	Alive no disease
37	3	55.9	131	Died from other cause
38	3	30	43	Died from disease
39	3	62	7	Died from disease
40	3	45.64	32	Died from disease
41	3	44	38	Died from disease
42	3	NA	NA	Alive no disease
43	3	47	27	Died from disease
44	3	62	35	Alive no disease
45	3	67	28	Alive no disease
46	3	56.59	30	Died from disease
47	3	47	8	Died from disease
48	3	79	103	Died from disease
49	3	69	34	NA
50	3	39	62	Died from other cause
51	3	54.58	23	Died from disease
52	3	78	49	Alive no disease
53	3	60	24	Died from disease
54	3	NA	NA	Alive no disease
55	3	44	81	Died from disease
56	3	39	46	Alive no disease
57	3	38.84	44	Died from disease
58	3	46	43	Alive no disease
59	3	45.38	194	Alive no disease
60	3	66.18	69	Died from disease
61	3	68	59	Died from other cause
62	3	38	18	Died from disease
63	3	39	168	Died from other cause
64	3	62	9	Died from disease
65	3	62	148	Died from other cause
66	3	78	15	Died from disease
67	3	69	34	NA
68	3	60	195	Died from disease
69	3	62	102	Died from disease
70	3	58	45	Died from disease
